# Socioeconomic differences in the use of ill-defined causes of death in 16 European countries

**DOI:** 10.1186/1471-2458-14-1295

**Published:** 2014-12-17

**Authors:** Ivana Kulhánová, Gwenn Menvielle, Matthias Bopp, Carme Borrell, Patrick Deboosere, Terje A Eikemo, Rasmus Hoffmann, Mall Leinsalu, Pekka Martikainen, Enrique Regidor, Maica Rodríguez-Sanz, Jitka Rychtaříková, Bogdan Wojtyniak, Johan P Mackenbach

**Affiliations:** Department of Public Health, Erasmus Medical Center, P.O. Box 2040, 3000 CA Rotterdam, The Netherlands; INSERM, UMR_S 1136, Pierre Louis Institute of Epidemiology and Public Health, 75013 Paris, France; Sorbonne Universités, UPMC Univ Paris 06, UMR_S 1136, Pierre Louis Institute of Epidemiology and Public Health, 75013 Paris, France; Institute of Social and Preventive Medicine, University of Zürich, Zürich, Switzerland; Agència de Salut Pública de Barcelona, Barcelona, Spain; Department of Sociology, Vrije Universiteit Brussel, Brussels, Belgium; Department of Sociology and Political Science, Norwegian University of Science and Technology, Trondheim, Norway; Stockholm Centre on Health of Societies in Transition, Södertörn University, Huddinge, Sweden; Department of Epidemiology and Biostatistics, National Institute for Health Development, Tallinn, Estonia; Department of Sociology, University of Helsinki, Helsinki, Finland; Department of Preventive Medicine and Public Health, Universidad Complutense de Madrid, Madrid, Spain; Department of Demography and Geodemography, Faculty of Science, Charles University in Prague, Prague, Czech Republic; Department-Centre for Monitoring and Analyses of Population Health Status and Health Care System, National Institute of Public Health – National Institute of Hygiene, Warsaw, Poland

**Keywords:** Mortality, Education, Ill-defined causes of death, Data quality, Europe

## Abstract

**Background:**

Cause-of-death data linked to information on socioeconomic position form one of the most important sources of information about health inequalities in many countries. The proportion of deaths from ill-defined conditions is one of the indicators of the quality of cause-of-death data. We investigated educational differences in the use of ill-defined causes of death in official mortality statistics.

**Methods:**

Using age-standardized mortality rates from 16 European countries, we calculated the proportion of all deaths in each educational group that were classified as due to “Symptoms, signs and ill-defined conditions”. We tested if this proportion differed across educational groups using Chi-square tests.

**Results:**

The proportion of ill-defined causes of death was lower than 6.5% among men and 4.5% among women in all European countries, without any clear geographical pattern. This proportion statistically significantly differed by educational groups in several countries with in most cases a higher proportion among less than secondary educated people compared with tertiary educated people.

**Conclusions:**

We found evidence for educational differences in the distribution of ill-defined causes of death. However, the differences between educational groups were small suggesting that socioeconomic inequalities in cause-specific mortality in Europe are not likely to be biased.

**Electronic supplementary material:**

The online version of this article (doi:10.1186/1471-2458-14-1295) contains supplementary material, which is available to authorized users.

## Background

Cause-of-death statistics are an important source of information for epidemiological research and policy decisions. Their reliability is essential, not only for assessing trends and variations in average population health, but also for assessing the magnitude of inequalities in health between population groups. Indeed, many studies of health inequalities make extensive use of cause-specific mortality data [1–4], and it is therefore important to ensure that there are no differences between socioeconomic groups in the quality of cause-of-death information.

The proportion of deaths from ill-defined conditions is one of the commonly used indicators for the quality of cause-of-death data [5–8]. Deaths should be classified as due to ill-defined conditions only in a few cases when the real cause of death cannot be determined. However, in practice, deaths may also be classified as ill-defined when the certifying physician has insufficient knowledge of the disease(s) causing death, and/or has not completed the death certificate properly. It is likely that ill-defined causes of death hide important pathologies, and a high proportion of ill-defined causes of death may therefore lead to an underestimation of the mortality rates from well-defined causes of death, such as ischemic heart disease (IHD), suicide or injuries [8–11]. On the other hand, a very low proportion of ill-defined conditions does not necessarily imply a high quality of cause-of-death information, because it does not exclude other forms of misclassification such as a tendency to over-report one specific cause of death (e.g., cardiovascular disease) at the expense of another [12].

Previous studies have shown that deaths from ill-defined conditions are more common in ethnic minorities [9], among old people living alone and in very marginal population groups, such as homeless people [13, 14]. Although a Dutch study reported higher mortality due to ill-defined conditions in low-income boroughs of Amsterdam [15], studies investigating socioeconomic differences in the proportion of ill-defined causes of death are rare and were not conducted in such extent as our study does. Such inequalities may occur, for example, if lower socioeconomic groups have less access to good quality health care [16, 17] and, as a consequence, die under circumstances in which their diagnosis is less well-established than is normally the case for patients with a higher socioeconomic position.

Socioeconomic differences in the proportion of ill-defined causes of death may lead to under- or overestimation of socioeconomic differences in well-defined causes of death. The aim of the present study was therefore to examine whether there are educational differences in the proportion of ill-defined causes of death among men and women in 16 European populations and to investigate if these differences harm the socioeconomic differences in mortality from specific causes of deaths, especially ischemic heart disease or suicide.

## Methods

We analysed mortality data from 16 European populations as collected and harmonized in the EURO-GBD-SE project [18]. Data come from longitudinal (Finland, Sweden, Norway, Denmark, England and Wales, Netherlands, Belgium, France, Switzerland, Austria, the Basque Country, Madrid, Turin, Tuscany), repeated cross-sectional (Barcelona) or cross-sectional unlinked (Hungary, Czech Republic, Poland, Estonia) studies in national, regional or urban populations in the time period between 1998 and 2007 (Additional file 1: Table S1 in the supplementary online material). We combined all Spanish and all Italian datasets to ensure adequate number of deaths.

Completed education was categorized into three groups according to the International Standard Classification of Education (ISCED): less-than-secondary education (ISCED 0, 1, 2; ‘low’), secondary education (ISCED 3, 4; ‘mid’) and tertiary education (ISCED 5, 6; ‘high’). The share of individuals with unknown education was in most populations below 2.3% except in France (6.0%) and Switzerland (6.1%). These individuals were excluded from the analyses. Ill-defined causes of death were defined as codes 780–799 (“Symptoms, signs and ill-defined conditions”) or R00–R99 (“Symptoms, signs and abnormal clinical and laboratory findings, not elsewhere classified”), according to respectively the ninth or tenth revision of the International Classification of Diseases. Examples of specific entities within this chapter are ‘sudden death’, ‘senility’ or ‘old age’.

Analyses were conducted by country, sex and education for the age range 30–79. The proportion of ill-defined causes of death was computed as the share of the age-standardized mortality rate (ASMR) for ill-defined conditions on the all-cause ASMR. We used direct standardization with European Standard Population as standard [19]. We performed Chi-square tests of independence to assess if the proportion of ill-defined causes of death differed by educational group [20]. All tests were performed at the 5% significance level.

Further, we conducted a sensitivity analysis in which we estimated to what extent any misclassification of well-defined causes of death as ill-defined condition may affect socioeconomic inequalities in those well-defined causes. We used IHD (ICD-9: 410–414, ICD-10: I20–I25) and suicide (ICD-9: E950–959; ICD-10: X60–X84, Y87.0) as examples for the misclassification. We conducted the sensitivity analysis by adding 50% (respectively 20%) of deaths from ill-defined conditions to IHD deaths (resp. suicide deaths) in each educational group. We assessed relative inequalities as relative risks using Poisson regression. Subsequently, we compared the ranking of socioeconomic inequalities in IHD and suicide mortality between European countries before and after the redistribution. The ranking was calculated by the Spearman correlation.

## Results

**Figure 1:**
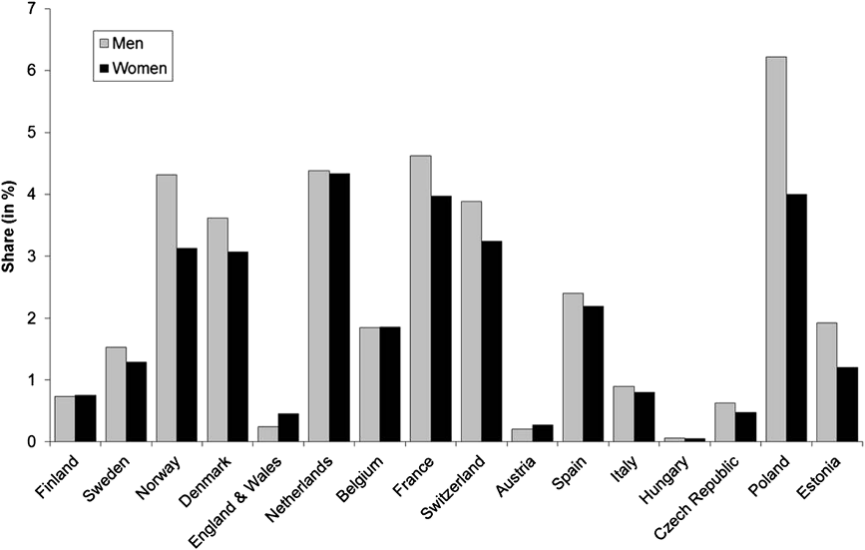
**Share of ASMR from ill-defined conditions on all-cause ASMR (in %) by country and sex, 30–79 years.**

The proportion of ill-defined causes of death varied across European countries, but without any clear geographical pattern (Figure 1). The proportion ranged from 0.1% in Hungary to 6.2% in Poland among men, and from 0.05% in Hungary to 4.3% in the Netherlands among women. For both men and women, proportions of ill-defined causes of death lower than 1% were found in Finland, England and Wales, Scotland, Austria, Italy, Hungary, Czech Republic and Lithuania. Proportions of ill-defined causes of death higher than 3% were observed in Norway, Denmark, Netherlands, France, Switzerland and Poland.

The distribution of ill-defined causes of death differed by educational level in Denmark, England and Wales, Belgium, Hungary, Czech Republic, Poland and Estonia among men, and in Poland among women with the tendency of a higher proportion among low educated, and in Switzerland (men and women) and Italy (men) with the tendency of a higher proportion among high educated (Table 1). Absolute differences between the low and high educated are, however, small: generally less than one percentage point, with exception of Polish men among whom the difference is 2.9%-points.

**Table 1:** **Number of deaths from ill-defined conditions (D) and share of ASMR from ill-defined conditions on all-cause ASMR (in %) by country, sex and educational level, 30–79 years**

Country	Men							Women						
	Low education		Mid education		High education		P-value ^a^	Low education		Mid education		High education		P-value ^a^
	D	%	D	%	D	%		D	%	D	%	D	%	
**Finland**	308	0.7	221	0.8	108	0.8	0.822	161	0.8	99	0.8	82	1.2	0.071
**Sweden**	879	1.6	733	1.6	224	1.6	0.779	669	1.3	371	1.3	123	1.4	0.655
**Norway**	808	4.2	983	4.4	285	4.8	0.211	553	2.9	454	3.3	101	3.6	0.058
**Denmark**	1,530	3.9	944	3.4	282	3.2	0.000	1,273	3.1	405	3.1	157	3.0	0.928
**England & Wales**	16	0.4	*	0.1	0	0.0	0.000	21	0.4	*	0.1	*	1.0	0.071
**Netherlands**	120	4.0	102	4.6	55	5.1	0.218	107	4.1	47	4.9	14	3.7	0.597
**Belgium**	767	2.0	156	1.7	107	1.6	0.038	545	1.9	91	1.8	58	1.8	0.820
**France**	202	4.5	118	4.7	36	6.9	0.121	123	4.1	37	4.2	11	5.0	0.835
**Switzerland**	528	3.5	1,074	3.8	498	5.0	0.000	566	3.1	538	3.3	114	5.2	0.000
**Austria**	21	0.3	24	0.2	*	0.1	0.571	42	0.3	16	0.3	*	0.2	0.789
**Spain**	1,463	2.5	292	2.4	268	2.5	0.753	863	2.3	99	2.2	75	2.0	0.427
**Italy**	99	0.8	28	0.8	28	1.9	0.017	77	0.8	18	0.9	*	0.4	0.198
**Hungary**	100	0.1	35	0.1	*	0.0	0.000	51	0.1	10	0.0	*	0.0	0.096
**Czech Republic**	1036	0.7	159	0.5	69	0.6	0.012	540	0.5	83	0.4	27	0.8	0.109
**Poland**	13,532	7.8	13,461	5.7	1,113	4.9	0.000	7,210	4.7	3,476	3.7	387	4.0	0.000
**Estonia**	350	2.1	306	1.9	44	1.4	0.017	199	1.2	104	1.1	11	0.7	0.208

^a^Chi-square test for the difference across educational groups in the share of ASMR from ill-defined conditions on all-cause ASMR.

*D < = 5; Although we cannot present numbers smaller than 5 in the table, we did use them in the analysis.

Although in general the proportion of ill-defined causes of death on total mortality was higher among lower educated, we observed a tendency for a higher proportion among high educated in Finland (women), Norway, Netherlands (men), France, Switzerland and Italy (men). This is the case for countries which combine relatively low total mortality rate with relatively high mortality rate from ill-defined conditions among high educated individuals.

Based on the sensitivity analysis, relative inequalities in IHD and suicide mortality changed considerably only among men in Poland after the redistribution of the ill-defined causes of death (Tables 2 and 3). These redistributions did not change the rank order for relative inequalities in IHD mortality (rho: men, women = 0.956) and suicide mortality (rho: men = 0.982; women = 0.970) among the European countries investigated.

**Table 2:** **Relative risks (RR) of ischemic heart disease (IHD) mortality before and after a 50%-redistribution of ill-defined causes of death, with corresponding 95% confidence intervals (CI), by country and educational level, men and women, 30–79 years**

Country	Education	MEN				WOMEN			
		IHD		IHD after redistribution		IHD		IHD after redistribution	
		RR	95%-CI	RR	95%-CI	RR	95%-CI	RR	95%-CI
**Finland**	**low**	2.16	(2.07–2.25)	2.15	(2.06–2.24)	2.66	(2.43–2.92)	2.57	(2.36–2.81)
	**mid**	1.67	(1.59–1.76)	1.67	(1.59–1.75)	1.82	(1.64–2.01)	1.76	(1.59–1.94)
	**high**	1		1		1		1	
**Sweden**	**low**	2.10	(2.01–2.19)	2.08	(1.99–2.17)	2.78	(2.56–3.02)	2.68	(2.48–2.89)
	**mid**	1.57	(1.50–1.65)	1.56	(1.50–1.63)	1.95	(1.79–2.12)	1.89	(1.74–2.05)
	**high**	1		1		1		1	
**Norway**	**low**	2.49	(2.30–2.68)	2.41	(2.24–2.58)	3.05	(2.60–3.57)	2.75	(2.39–3.16)
	**mid**	1.70	(1.57–1.83)	1.66	(1.54–1.78)	1.79	(1.53–2.11)	1.68	(1.46–1.94)
	**high**	1		1		1		1	
**Denmark**	**low**	1.90	(1.78–2.03)	1.94	(1.82–2.06)	2.32	(2.05–2.63)	2.21	(1.98–2.47)
	**mid**	1.54	(1.43–1.65)	1.55	(1.45–1.65)	1.54	(1.34–1.76)	1.50	(1.33–1.69)
	**high**	1		1		1		1	
**England & Wales**	**low**	1.63	(1.39–1.91)	1.64	(1.40–1.93)	2.62	(1.93–3.56)	2.52	(1.87–3.40)
	**mid**	1.26	(1.05–1.51)	1.26	(1.05–1.51)	1.55	(1.08–2.22)	1.48	(1.04–2.10)
	**high**	1		1		1		1	
**Netherlands**	**low**	2.11	(1.72–2.59)	1.97	(1.63–2.37)	2.81	(1.72–4.61)	2.51	(1.65–3.82)
	**mid**	1.54	(1.24–1.90)	1.45	(1.20–1.76)	1.66	(0.98–2.82)	1.60	(1.02–2.51)
	**high**	1		1		1		1	
**Belgium**	**low**	1.82	(1.69–1.97)	1.85	(1.72–1.99)	2.23	(1.90–2.63)	2.16	(1.86–2.51)
	**mid**	1.36	(1.23–1.49)	1.37	(1.25–1.50)	1.49	(1.24–1.81)	1.47	(1.23–1.75)
	**high**	1		1		1		1	
**France**	**low**	2.15	(1.58–2.93)	2.00	(1.54–2.60)	3.65	(1.60–8.31)	2.69	(1.47–4.91)
	**mid**	1.65	(1.20–2.28)	1.54	(1.17–2.02)	1.66	(0.68–4.01)	1.49	(0.78–2.84)
	**high**	1		1		1		1	
**Switzerland**	**low**	1.94	(1.82–2.08)	1.88	(1.76–1.99)	2.21	(1.84–2.65)	1.82	(1.56–2.13)
	**mid**	1.42	(1.33–1.50)	1.38	(1.30–1.46)	1.42	(1.18–1.71)	1.22	(1.04–1.42)
	**high**	1		1		1		1	
**Austria**	**low**	1.62	(1.46–1.80)	1.63	(1.47–1.80)	1.88	(1.47–2.41)	1.88	(1.47–2.41)
	**mid**	1.49	(1.35–1.64)	1.49	(1.35–1.65)	1.35	(1.05–1.74)	1.36	(1.06–1.74)
	**high**	1		1		1		1	
**Spain**	**low**	1.24	(1.16–1.32)	1.26	(1.19–1.34)	1.55	(1.31–1.83)	1.56	(1.35–1.82)
	**mid**	1.11	(1.02–1.21)	1.11	(1.03–1.20)	1.19	(0.96–1.48)	1.23	(1.02–1.49)
	**high**	1		1		1		1	
**Italy**	**low**	1.38	(1.19–1.59)	1.33	(1.16–1.53)	1.20	(0.91–1.58)	1.24	(0.95–1.63)
	**mid**	1.08	(0.91–1.28)	1.04	(0.88–1.23)	0.82	(0.59–1.14)	0.87	(0.63–1.20)
	**high**	1		1		1		1	
**Hungary**	**low**	2.46	(2.38–2.56)	2.47	(2.38–2.56)	2.41	(2.23–2.60)	2.41	(2.24–2.60)
	**mid**	1.25	(1.20–1.31)	1.25	(1.20–1.31)	1.22	(1.13–1.33)	1.22	(1.13–1.33)
	**high**	1		1		1		1	
**Czech Republic**	**low**	2.90	(2.78–3.02)	2.90	(2.78–3.03)	3.26	(2.91–3.65)	3.17	(2.84–3.55)
	**mid**	1.56	(1.49–1.64)	1.56	(1.49–1.63)	1.86	(1.66–2.10)	1.82	(1.62–2.04)
	**high**	1		1		1		1	
**Poland**	**low**	1.98	(1.92–2.05)	2.21	(2.15–2.28)	2.49	(2.32–2.66)	2.46	(2.32–2.62)
	**mid**	1.91	(1.85–1.97)	1.96	(1.90–2.02)	1.97	(1.84–2.11)	1.92	(1.80–2.04)
	**high**	1		1		1		1	
**Estonia**	**low**	2.31	(2.14–2.49)	2.34	(2.17–2.52)	2.50	(2.24–2.80)	2.53	(2.26–2.83)
	**mid**	1.92	(1.77–2.07)	1.93	(1.79–2.09)	1.86	(1.66–2.09)	1.88	(1.68–2.11)
	**high**	1		1		1		1

**Table 3:** **Relative risks (RR) of suicide mortality before and after a 20%-redistribution of ill-defined causes of death, with corresponding 95% confidence intervals (CI), by country and educational level, men and women, 30–79 years**

Country	Education	MEN				WOMEN			
		Suicide		Suicide after redistribution		Suicide		Suicide after redistribution	
		RR	95%-CI	RR	95%-CI	RR	95%-CI	RR	95%-CI
**Finland**	**low**	2.08	(1.88–2.31)	2.07	(1.88–2.29)	1.68	(1.42–1.98)	1.64	(1.40–1.93)
	**mid**	1.76	(1.60–1.95)	1.75	(1.59–1.94)	1.40	(1.20–1.63)	1.37	(1.18–1.60)
	**high**	1		1		1		1	
**Sweden**	**low**	1.88	(1.70–2.09)	1.85	(1.68–2.05)	1.33	(1.15–1.55)	1.35	(1.17–1.56)
	**mid**	1.45	(1.31–1.60)	1.43	(1.30–1.58)	1.18	(1.04–1.35)	1.18	(1.04–1.34)
	**high**	1		1		1		1	
**Norway**	**low**	2.15	(1.79–2.59)	2.07	(1.77–2.42)	1.24	(0.95–1.62)	1.32	(1.05–1.66)
	**mid**	1.60	(1.37–1.87)	1.55	(1.35–1.78)	1.15	(0.93–1.43)	1.17	(0.96–1.42)
	**high**	1		1		1		1	
**Denmark**	**low**	1.82	(1.59–2.09)	1.92	(1.69–2.17)	1.17	(0.96–1.42)	1.29	(1.08–1.52)
	**mid**	1.49	(1.30–1.71)	1.51	(1.33–1.72)	0.97	(0.79–1.20)	1.05	(0.87–1.26)
	**high**	1		1		1		1	
**England & Wales**	**low**	2.08	(1.08–4.00)	2.19	(1.14–4.19)	0.97	(0.37–2.53)	0.93	(0.38–2.25)
	**mid**	1.34	(0.65–2.76)	1.35	(0.66–2.77)	0.48	(0.13–1.59)	0.41	(0.12–1.36)
	**high**	1		1		1		1	
**Netherlands**	**low**	1.17	(0.75–1.84)	1.21	(0.82–1.76)	0.91	(0.46–1.77)	1.08	(0.60–1.92)
	**mid**	0.76	(0.47–1.21)	0.85	(0.57–1.25)	1.04	(0.54–2.01)	1.11	(0.62–2.00)
	**high**	1		1		1		1	
**Belgium**	**low**	1.75	(1.55–1.97)	1.78	(1.59–2.00)	0.96	(0.81–1.14)	1.00	(0.85–1.18)
	**mid**	1.43	(1.25–1.63)	1.43	(1.26–1.63)	0.91	(0.75–1.10)	0.93	(0.77–1.12)
	**high**	1		1		1		1	
**France**	**low**	3.24	(2.03–5.15)	2.80	(1.87–4.19)	2.35	(1.18–4.70)	2.16	(1.16–4.05)
	**mid**	2.46	(1.55–3.89)	2.15	(1.44–3.21)	1.43	(0.70–2.92)	1.42	(0.74–2.71)
	**high**	1		1		1		1	
**Switzerland**	**low**	1.67	(1.48–1.89)	1.63	(1.45–1.82)	1.08	(0.88–1.33)	1.03	(0.85–1.25)
	**mid**	1.40	(1.27–1.54)	1.35	(1.24–1.48)	1.10	(0.91–1.33)	1.02	(0.86–1.22)
	**high**	1		1		1		1	
**Austria**	**low**	2.30	(1.78–2.97)	2.31	(1.79–2.97)	1.38	(0.89–2.15)	1.38	(0.89–2.14)
	**mid**	1.78	(1.40–2.26)	1.78	(1.41–2.26)	0.95	(0.61–1.47)	0.95	(0.62–1.48)
	**high**	1		1		1		1	
**Spain**	**low**	2.05	(1.71–2.47)	1.91	(1.63–2.23)	1.50	(1.13–2.00)	1.53	(1.18–1.97)
	**mid**	1.39	(1.12–1.72)	1.32	(1.10–1.59)	1.55	(1.13–2.13)	1.52	(1.14–2.03)
	**high**	1		1		1		1	
**Italy**	**low**	1.40	(0.91–2.17)	1.26	(0.85–1.87)	0.83	(0.45–1.50)	0.91	(0.51–1.62)
	**mid**	1.42	(0.89–2.27)	1.25	(0.82–1.92)	0.92	(0.48–1.75)	1.00	(0.54–1.85)
	**high**	1		1		1		1	
**Hungary**	**low**	6.16	(5.48–6.93)	6.17	(5.49–6.94)	2.90	(2.38–3.54)	2.90	(2.38–3.54)
	**mid**	2.63	(2.34–2.97)	2.64	(2.34–2.97)	1.73	(1.41–2.12)	1.73	(1.41–2.12)
	**high**	1		1		1		1	
**Czech Republic**	**low**	2.49	(2.23–2.79)	2.52	(2.26–2.81)	1.67	(1.29–2.16)	1.64	(1.28–2.10)
	**mid**	1.23	(1.08–1.39)	1.23	(1.08–1.39)	1.36	(1.04–1.78)	1.31	(1.01–1.70)
	**high**	1		1		1		1	
**Poland**	**low**	5.80	(2.23–6.44)	5.20	(4.78–5.65)	2.37	(1.97–2.85)	2.39	(2.07–2.76)
	**mid**	2.98	(2.69–3.30)	2.77	(2.55–3.01)	1.65	(1.38–1.97)	1.66	(1.44–1.91)
	**high**	1		1		1		1	
**Estonia**	**low**	3.14	(2.52–3.91)	3.17	(2.57–3.91)	2.26	(1.50–3.40)	2.40	(1.64–3.54)
	**mid**	2.25	(1.82–2.79)	2.27	(1.85–2.79)	1.56	(1.07–2.27)	1.64	(1.15–2.36)
	**high**	1		1		1		1	

## Discussion

This study had a broad geographical scope and included countries with different educational systems and different cause-of-death certifying and coding practices. Although we put much effort in harmonizing the data, there are some methodological issues that should be addressed.

First, foreigners and people born outside mainland were excluded from the Swiss and French dataset, respectively. If cause-of-death certification is more incomplete among foreigners [21], this will have led to an underestimation of the proportion of ill-defined causes of death in these countries. Spain and Italy were represented by cities or regions. If cause-of-death information is more complete in these urban areas [7], this will again have led to an underestimation of the proportion of ill-defined causes of death in these countries. Whether this may also have had an impact on our results for educational inequalities is, however, difficult to assess.

Second, people with unknown education were excluded from the analyses. The share of ill-defined causes of death was higher among people with unknown education compared with those with primary education. However, due to the small percentage of unknown education in the mortality datasets, our conclusions do not change when combining unknown education with primary education (results not shown).

Third, some datasets had a cross-sectional unlinked design, whereas other consisted of census-linked mortality follow-up studies. It has been shown that mortality inequalities based on unlinked datasets are likely to suffer from the numerator-denominator bias [13, 22], observed when information on education comes from death certificates for the deceased (the numerator), and from census for the population (the denominator) [23]. Education misreporting has been found larger for deaths from ill-defined conditions [22]. This could spuriously produce inequalities in mortality from ill-defined conditions in countries with cross-sectional unlinked designs. Although all four countries showed statistically significant results, only Poland had an exceptionally high proportion of ill-defined causes of death, which has been reported previously [7, 24].

Forth, there is a large variation in sample sizes between European countries. This might have affected the power to clearly detect differences in the proportion of ill-defined causes of death by educational level. In some countries, these differences were statistically significant probably due to the extremely small number of cases in one of the three educational categories. The variations in sample sizes might have also affected differences observed between men and women. Whereas socioeconomic differences in the proportion of ill-defined causes of death were found in about half of the countries among men, these differences were detected only in two countries among women. These gender differences may be, at least partly, due to the differences in sample sizes as the number of deaths was much lower among women than among men.

As mentioned in the introduction, ill-defined causes of death may hide important well-defined causes of death. Autopsy may play an important role in order to identify the correct well-defined cause of death. It has been reported in Barcelona that after forensic tests only 28% of ill-defined causes of death remained ill-defined, the rest being redistributed in other specific causes of death, mainly diseases of circulatory system and to a lesser extent suicide [25]. The percentage of autopsies varies considerably between European countries. In 2000s, this percentage conducted was less than 10% in Norway, Denmark, Netherlands and Switzerland, about 15% in Sweden, and 30% or more in Finland, Austria, Hungary, Czech Republic, Lithuania and Estonia, and not available in Belgium, France, UK, Spain, Italy and Poland [24]. Our findings suggest that the lower the percentage of autopsies the higher the proportion of ill-defined causes of death. Except Poland, for which we do not have information about the autopsy rate, Norway, Denmark, Netherlands and Switzerland are countries with the lowest autopsy rate in Europe but with one of the highest percentages of ill-defined causes of death. This negative correlation between the autopsy rate and the proportion of ill-defined causes of death has been reported previously [26].

The literature suggests that IHD [27, 28] or suicide [11, 29] may be misclassified as ill-defined causes of death. IHD could be misclassified because deaths from myocardial infarctions may occur suddenly, and when due to cardiac arrhythmia are not detectable even with autopsy. Suicide could be misclassified due to religious taboos, cultural norms and social stigma or due to lack of evidence [30].

## Conclusions

We found educational differences in the proportion of ill-defined causes of death in several European countries. However the percentage difference was not large enough to impact educational inequalities in well-defined causes of death after a redistribution of ill-defined causes of death. In other words, if the proportion of ill-defined conditions remains small, any redistribution of these causes of death will have only a negligible impact on socioeconomic inequalities in well-defined causes of death. This highlights the importance of keeping the proportion of ill-defined causes of death in the national mortality statistics at a possible minimum if we want to get meaningful statistics from cause of death data. Although there may be other forms of misclassification, our results suggest that findings from previous studies documenting socioeconomic inequalities in cause-specific mortality in Europe are not likely to be biased by differences in the quality of cause-of-death information.

## Electronic supplementary material

Additional file 1: Table S1: Mortality data sources. (PDF 42 KB)
