# Supplementary material for: Socioeconomic differences in the use of ill-defined causes of death in 16 European countries
Source: BMC Public Health. 2014 Dec 17;14:1295. doi: 10.1186/1471-2458-14-1295 (PMC4302075; doi:10.1186/1471-2458-14-1295)
Supplement: Supplementary file 1 — Additional file 1: Table S1: Mortality data sources. (PDF 42 KB) [file 12889_2014_7430_MOESM1_ESM.pdf]

Socioeconomic differences in the use of ill-defined causes of death in 16 European countries

Ivana Kulhánová[1,*], Gwenn Menvielle[2,3], Matthias Bopp[4], Carme Borrell[5], Patrick Deboosere[6], Terje A. Eikemo[1,7], Rasmus Hoffmann[1], Mall Leinsalu[8,9], Pekka Martikainen[10], Enrique Regidor[11], Maica Rodríguez-Sanz[5], Jitka Rychtaříková[12], Bogdan Wojtyniak[13], Johan P. Mackenbach[1]

[1] Department of Public Health, Erasmus MC, University Medical Center Rotterdam, P.O. Box 2040, 3000 CA Rotterdam, The Netherlands
[2] INSERM, UMR_S 1136, Pierre Louis Institute of Epidemiology and Public Health, 75013, Paris, France
[3] Sorbonne Universités, UPMC Univ Paris 06, UMR_S 1136, Pierre Louis Institute of Epidemiology and Public Health, 75013, Paris, France
[4] Institute of Social and Preventive Medicine, University of Zürich, Zürich, Switzerland
[5] Agència de Salut Pública de Barcelona, Barcelona, Spain
[6] Department of Sociology, Vrije Universiteit Brussel, Brussels, Belgium
[7] Department of Sociology and Political Science, Norwegian University of Science and Technology, Trondheim, Norway
[8] Stockholm Centre on Health of Societies in Transition, Södertörn University, Huddinge, Sweden
[9] Department of Epidemiology and Biostatistics, National Institute for Health Development, Tallinn, Estonia
[10] Department of Sociology, University of Helsinki, Helsinki, Finland
[11] Department of Preventive Medicine and Public Health, Universidad Complutense de Madrid, Madrid, Spain
[12] Department of Demography and Geodemography, Faculty of Science, Charles University in Prague, Prague, Czech Republic
[13] Department-Centre for Monitoring and Analyses of Population Health Status and Health Care System, National Institute of Public Health – National Institute of Hygiene, Warsaw, Poland

[*] Corresponding author: i.kulhanova@erasmusmc.nl

BMC PUBLIC HEALTH – ELECTRONIC SUPPLEMENTARY MATERIAL

Table S1: Mortality data sources

Population	Type of dataset	Period	Geographic coverage	Demographic coverage	Number of person-years#	Number of deaths#
**Finland**	longitudinal	2001–2007	national	20% of Finns are excluded (at random)	16,384,591	145,795
**Sweden**	longitudinal	2001–2006	national	whole population	29,781,132	214,300
**Norway**	longitudinal	2001–2006	national	whole population	12,309,468	83,343
**Denmark**	longitudinal	2001–2005	national	whole population	15,209,876	134,925
**England & Wales**	longitudinal	2001–2006	national	1% of the population	1,518,487	13,124
**Netherlands**	longitudinal	1998–2007	national	linkage based on the Labour Force Survey	1,802,289	9,875
**Belgium**	longitudinal	2004–2005	national	whole population	11,946,186	94,551
**France**	longitudinal	1999–2005	national	1% of the population, born outside France mainland excluded	1,626,611	12,270
**Switzerland**	longitudinal	2001–2005	national	Non-Swiss nationals excluded	16,448,691	92,656
**Austria**	longitudinal	2001–2002	national	whole population	4,872,243	39,690
**Barcelona**	repeated CS	2000–2006	city	whole population	7,387,813	52,444
**Basque**	longitudinal	2001–2006	region	whole population	6,722,857	46,277
**Madrid**	longitudinal	2001–2003	region	whole population	5,216,958	36,526
**Turin**	longitudinal	2001–2006	city	whole population	2,615,579	21,188
**Tuscany**	longitudinal	2001–2005	Florence, Leghorn, Prato	whole population	1,559,031	12,263
**Hungary**	CS unlinked	1999–2002	national	whole population	23,834,776	371,492
**Czech Republic**	CS unlinked	1999–2003	national	whole population	29,122,645	348,019
**Poland**	CS unlinked	2001–2003	national	whole population	62,078,814	726,093
**Estonia**	CS unlinked	1998–2002	national	whole population	3,873,395	61,918

# age range 30–79 years
CS = cross-sectional
